# POLYPHARMACY IN ASSISTED LIVING

**DOI:** 10.1093/geroni/igac059.992

**Published:** 2022-12-20

**Authors:** Elizabeth Galik

**Affiliations:** University of Maryland, Baltimore, Baltimore, Maryland, United States

## Abstract

The purpose of this study was to describe polypharmacy in assisted living settings, evaluate the factors that influence polypharmacy and the impact of polypharmacy on clinical outcomes. Baseline data from the study entitled, Dissemination and Implementation of Function Focused Care for Assisted Living Using the Evidence Integration Triangle (FFC-AL-EIT) was used. Total number of drugs taken daily among the 781 participants was 5.16 (SD=2.40) and over half (N=484, 62%) were exposed to polypharmacy. None of the predicted variables (age, gender, race, setting, diagnoses, and cognition) were associated with polypharmacy (Wald = .207, p=.65). Similarly, controlling for age, gender, race, setting, diagnoses, cognition, function, and physical activity, polypharmacy was not associated with falls, emergency room visits or hospitalizations. Factors not included in the data contributing to the high rate of polypharmacy in assisted living settings will be discussed and recommendations for further research and practice implications reviewed.

